# Does clomiphene citrate *versus* recombinant FSH in intrauterine insemination cycles differ in follicular development?

**DOI:** 10.5935/1518-0557.20220039

**Published:** 2023

**Authors:** Ankita Sethi, Neeta Singh, Garima Patel

**Affiliations:** 1 Department of Obstetrics and Gynaecology, All India Institute of Medical Sciences, New Delhi

Dear Editor

We read the recently published article in your journal with great interest (Tokgoz *et al*., 2021). As the reader, we would like to put forward some concerns based on the study analysis:

Study references quoted in introduction are more than 10 years old and no recent studies have been discussed.

Letrozole is a proven better ovulation induction agent as compared to clomiphene citrate (CC), so a third arm of letrozole should have been included, for better clinical implication of study. A recent study recommended letrozole as the drug of choice in infertile women undergoing ovulation induction and IUI, especially PCOS patients, since it has highest mono-follicular growth rate (Huang *et al*., 2018).

The indications of opting for CC arm *versus* rFSH arm needs to be mentioned.

Usually the starting dose of CC is 50 mg. Therefore, using 100 mg as the starting dose in the clomiphene arm in this study needs explanation.

In the methodology, titration of rFSH dose according to ovarian response is lacking.

The article includes 25% CC cycles and a 75% rFSH cycle, thus providing a heterogeneous group for analysis. The rationality of using rFSH more commonly needs clarification, especially when both clomiphene and letrozole give comparable results in normoresponder patients.

The cost effectiveness of using rFSH needs consideration, since it is an expensive drug, whereas oral and cheaper drugs like CC and letrozole have similar clinical outcome.

Since PCOS patients were also included in the study, some of them received rFSH as the ovulation agent. But there is no mention of established complications with rFSH use like multifollicular development, OHSS, cycle cancellation, or multiple pregnancy.

In Table 1, the baseline demographics and cycle characteristics were not similar between the two arms of the study, thus suggesting an unmatched population, making the clinical comparison futile.

Since the logistic regression analysis showed that bi-follicular development and endometrial thickness on the day of hCG significantly increased the odds of clinical pregnancy, this means Letrozole should have been the third arm for comparison as stimulation cycles with letrozole have better endometrial thickness/pattern (Wang *et al*., 2019) than CC, along with monofollicular development, and are substantially more cost effective, less cumbersome, painless than rFSH, especially in developing nations.
